# Supplementary figures and images for: Study of growth, metabolism, and morphology of Akkermansia muciniphila with an in vitro advanced bionic intestinal reactor
Source: BMC Microbiol. 2021 Feb 23;21:61. doi: 10.1186/s12866-021-02111-7 (PMC7901181; doi:10.1186/s12866-021-02111-7)

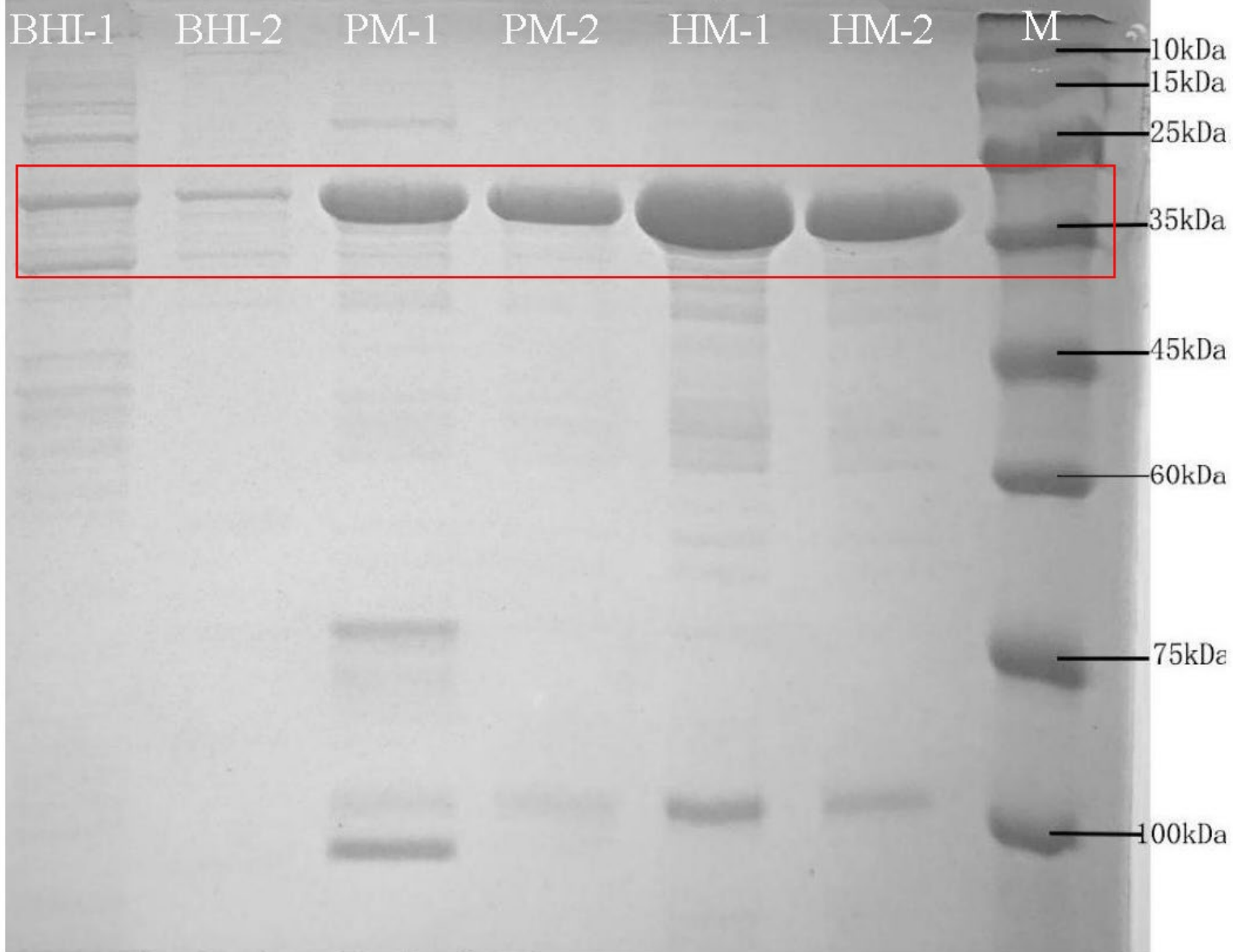

Supplement: Supplementary file 1 — Additional file 1: Figure S1. Amuc_1100 Western blot. In this figure, the brain heart infusion broth (BHI), porcine mucin (PM), human mucin (HM). [file 12866_2021_2111_MOESM1_ESM.pdf]
